# Inaugural editorial

**DOI:** 10.1007/s44216-022-00002-w

**Published:** 2022-10-18

**Authors:** Yongnian Zheng

**Affiliations:** 1 Institution of Politics and Economics, Shanghai Jiao Tong University, Shanghai, China; 2 The Institute for International Affairs (IIA), The Chinese University of Hong Kong, Shenzhen, China

The rise of Asia has been one of the most important trends reshaping global politics and the economy in the past few decades. When Japan completed its reindustrialization in merely two decades after the Second World War and joined the club of developed countries in the 1970s, it was viewed as an exception among Asian countries; at that time, few would have envisioned “a rising Asia.” Then came the Four Asian Tigers of Hong Kong, Singapore, South Korea and Chinese Taiwan, which started to undergo rapid economic development in the early 1960s and had developed into high income economies by the early 2000s. However, the real game changer arrived when China opened its door to the world economy and embarked on its journey of market-oriented reform in 1978. After over 40 years of unprecedented economic growth in speed and scale, the size of the Chinese economy increased by 40-fold from 1978 to 2020. China became the second largest economy in the world in 2010 and is expected to be the largest in the next decade. With a GDP per capita of 12.6 thousand US dollars in 2021, China is on the cusp of becoming a high-income economy. The sheer size of China as the most populous nation in the world compounds the historic significance of its economic transformation, which also puts Asia firmly on the map in regard to relative global economic power.

Asia’s growth miracles are not limited to East Asian countries. With a population comparable in size with China but a much younger demographic structure, India has emerged as one of the fastest growing economies over the last two decades, and it is on track to become the third largest economy in the world in the next ten years, according to several forecasts. Southeast Asian countries such as Malaysia, Indonesia, Vietnam and the Philippines have shown great potential in recent years for becoming regional economic powerhouses. The resource-rich countries in West Asia and Central Asia also have bright prospects as more structural reforms are implemented to boost development by embracing digitization and investing in green technologies. The growth momentums generated and sustained by various groups of countries in Asia have catapulted the region to new heights as a vital engine of global growth while lifting hundreds of millions of people out of poverty. Asia as a whole now produces approximately 40% of world GDP in nominal terms, while North America and Europe together account for slightly over 50%. Measured by purchasing power parity, Asia’s economic output has already eclipsed that of North America and Europe, and soon it will be greater than the rest of the world combined.

With a population of 4.7 billion in 2021, Asia is home to approximately 60% of humanity. While such a fact further accentuates the importance of the region, it also helps to put in perspective Asia’s success hitherto achieved in economic development. In terms of per capita performance, Asia is still below the world average in many aspects. Except for very few economies, such as Japan and the Four Asian Tigers, the vast majority of Asia is still composed of developing countries, each facing a unique set of internal and external issues. Asia has indeed been rising. However, the so-called “Asian Century” has not yet arrived, nor is its future realization preordained. Challenges abound for these countries to maintain a steady pace of development, especially in a world that has experienced tremendous changes over the past few years, such as the exacerbation of nationalism and the retreat from globalization.

Both the COVID-19 pandemic and the war in Ukraine are salient examples of events, expected or otherwise, which carry serious ramifications for the global political economy. It was not that long ago that many political commentators in Europe believed that large-scale interstate wars among European countries were memories of a distant past relegated to the dustbins of history. Now, after having enjoyed over 70 years of relative peace, the people in Europe are bearing the brunt of the immediate impact of the ongoing conflicts and are lamenting over the “return of tragedy” to the continent. Some even opine that the pandemic and the war together are marking the end of the golden age of globalization, while others maintain that globalization is here to stay despite the disruptions caused by these events, although the trade and investment relationships among countries and across regions may experience some reshuffling. In either case, it is unlikely that the mode of globalization as we know it in the past 30 years will return to business as usual when the pandemic and the war finally end.

We therefore are at a critical juncture in modern history. The evolving landscape of the global political economy calls for the deep and systematic exploration of the multiplicity of interconnected issues engendered by and accompanying such changes from a plurality of perspectives. I am thrilled and honored to introduce the Asian Review of Political Economy (ARPE), the first academic journal established in response to the growing demand for high-quality studies in an era witnessing rising challenges to globalization as well as dramatic changes in the geopolitical configuration of the Asia Pacific and beyond. By focusing on the political economy of Asian economies and the changing political economy beyond the region, ARPE distinguishes itself from the core focus areas of existing journals on the region and those on political economy in general.

The mission of ARPE is to promote a comprehensive understanding of the political economy of the Asian region in the context of a world that is experiencing profound changes. With rigorous editorial screening and assessment processes, it publishes original research works in all traditions of political economy, particularly those with a focus on the political economy in this region. The journal welcomes both quantitative and qualitative studies as well as theory-driven and policy-oriented research on a wide range of political economy issues, including but not limited to economic growth and development, income and wealth distribution, finance, trade, investment, industry and technology, as well as contemporary world politics and emerging global security challenges.

In addition to original research articles, ARPE will also publish reviews, perspectives and methodologies that are relevant to the scope covered by the journal, while special issues will be published in a timely manner to embrace new events and emerging trends that bring about significant impact. With collective efforts from the research community, our aim is to build ARPE into a flagship journal and a platform for promoting international academic exchange and worldwide knowledge dissemination in research on the political economy of the Asia Pacific and beyond. Over time, in addition to becoming a leading and ground-breaking forum for intellectual and policy debates, ARPE will also serve as a perennial archive of top-quality scholarly works available for the academic community and the general public at large.

As the Editor-in-Chief, I would like to take this opportunity to express my sincerest gratitude to all the members of the ARPE Editorial Board. Without their passion, volunteerism and professionalism, the successful launch of ARPE would not be possible. With the constant and rapid development of ARPE, more editors are much needed, especially those with expertise in relevant interdisciplinary fields and the latest frontiers. If you are interested in becoming an editor for ARPE, please contact me. Last, but most importantly, I wish to close this inaugural editorial by inviting you to join our community by submitting your exciting academic findings to ARPE as well as accepting invitations to provide reviews. Driven by passion and commitment, with your great support, we will develop ARPE into a highly influential international journal with a global impact in this very import intersection of multidisciplinary studies, where intellectual sparks collide and new ideas can germinate into truly innovative and creative solutions for the multitude of problems and challenges confronting humanity today.

## Data Availability

Not applicable.

